# Antegrade partial stent-in-stent deployment through dual endosonographically-created routes for recurrent bile duct cancer after right hepatectomy with hepaticojejunostomy

**DOI:** 10.1055/a-2884-1560

**Published:** 2026-06-12

**Authors:** Haruo Miwa, Shotaro Tsunoda, Yuto Matsuoka, Ritsuko Oishi, Yuichi Suzuki, Hiromi Tsuchiya, Shin Maeda

**Affiliations:** 1 Gastroenterological Center 26437 Yokohama City University Medical CenterYokohamaJapan; 2 Department of GastroenterologyYokohama City University Graduate School of MedicineYokohamaKanagawaJapan

Biliary drainage after hepaticojejunostomy can be challenging, particularly when recurrent tumor results in isolation of segmental intrahepatic bile ducts. Endoscopic ultrasound-guided hepaticogastrostomy (EUS-HGS) has been increasingly used as an alternative drainage approach in such patients. 1​ ​2​ However, the antegrade deployment of multiple stents through endosonographically created routes (ESCRs) has not been standardized. 3​ 4​ ​5​

A 60-year-old man who had undergone right hepatectomy with hepaticojejunostomy for bile duct cancer was admitted with cholangitis due to a hepaticojejunostomy stricture ( Fig. 1 ). EUS-HGS was initially performed via B2, and a 7-Fr dedicated plastic stent was placed. Because cholangitis subsequently developed in the isolated B3, an additional EUS-HGS was performed; however, recurrent cholangitis persisted. Biopsy from the B2 ESCR revealed adenocarcinoma, confirming local recurrence ( Fig. 2 ). To control refractory cholangitis and allow systemic chemotherapy, antegrade partial stent-in-stent deployment via dual ESCRs was attempted ( Fig. 3 ). First, a landmark guidewire was advanced alongside the B2 plastic stent into the jejunal limb and left in place. After reinsertion of the duodenoscope, a guidewire was placed alongside the B3 plastic stent. An 8-mm, 8-cm uncovered self-expandable metal stent (Niti-S Large Cell SR Slim Delivery, Taewoong Medical, Seoul, Korea) was deployed in an antegrade manner from B3 to the jejunal limb. A 7-Fr plastic stent (Through & Pass Type IT, Gadelius Medical, Tokyo, Japan) was placed in the B3 ESCR. The guidewire was then introduced through the B2 ESCR alongside the landmark guidewire and advanced through a mesh cell of the first metal stent. A second identical uncovered metal stent was then deployed in a partial stent-in-stent fashion. Finally, a 7-Fr plastic stent was left in the B2 ESCR ( Fig. 4 , Video 1 ). Transient fever resolved conservatively, and systemic chemotherapy was initiated.

**Fig. 1:**
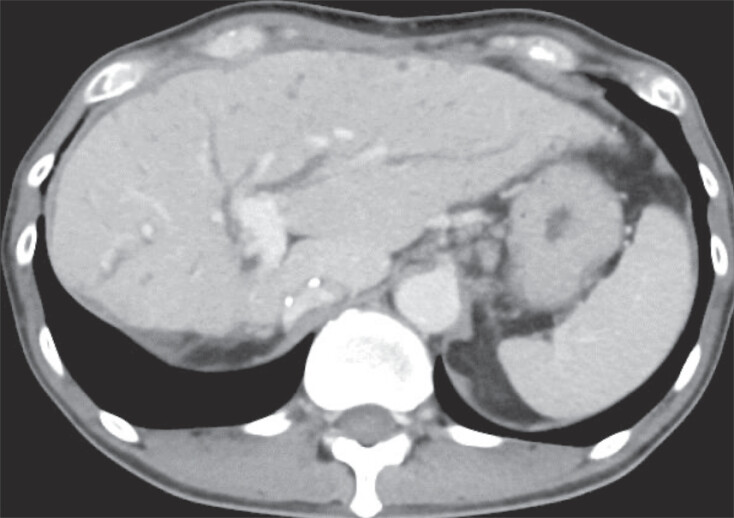
A computed tomography image shows dilated intrahepatic bile ducts.

**Fig. 2:**
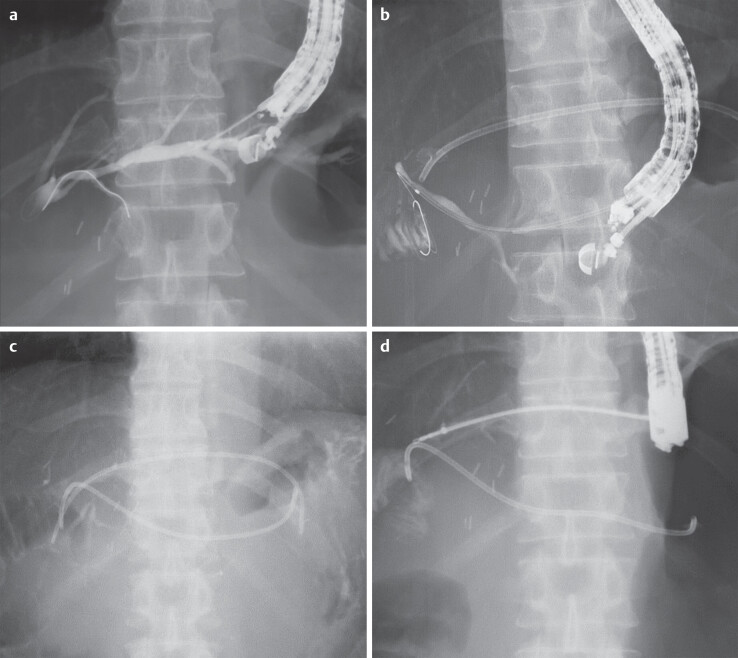
Fluoroscopic images during the treatment course. ( **a** ) Endoscopic ultrasound-guided hepaticogastrostomy is performed via B2. ( **b** ) Additional endoscopic ultrasound-guided hepaticogastrostomy is performed via B3. ( **c** ) Two plastic stents are placed through the dual endosonographically created routes (ESCRs) across the hepaticojejunostomy. ( **d** ) Biopsy through the B2 ESCR reveals adenocarcinoma, confirming local recurrence.

**Fig. 3:**
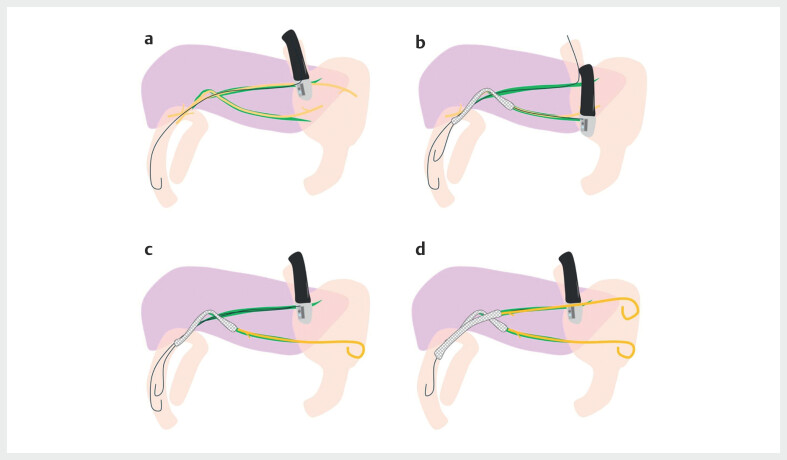
Schematic illustration of antegrade partial stent-in-stent deployment. ( **a** ) A landmark guidewire is advanced alongside the B2 plastic stent. ( **b** ) A self-expandable metal stent is deployed from B3 to the jejunal limb. ( **c** ) The working guidewire is inserted through the B2 ESCR and advanced through a mesh cell of the first metal stent. ( **d** ) A second metal stent is deployed in a partial stent-in-stent fashion. Plastic stents are placed in both ESCRs to preserve access for reintervention.

**Fig. 4:**
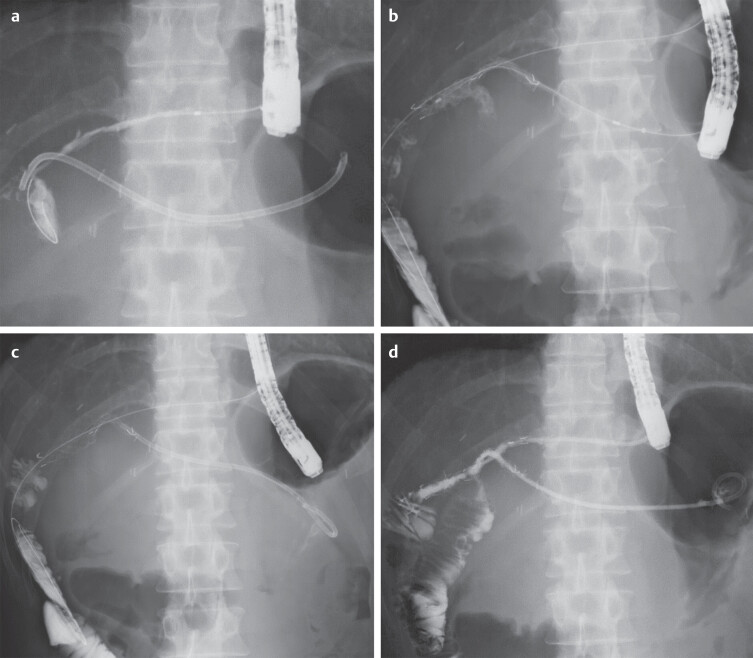
Fluoroscopic images of antegrade partial stent-in-stent deployment. ( **a** ) A landmark guidewire is advanced alongside the B2 plastic stent. ( **b** ) An 8 mm 8 cm, self-expandable metal stent is deployed from the B3 to the jejunal limb. ( **c** ) The working guidewire is advanced through a mesh cell of the first metal stent. ( **d** ) After deployment of the second metal stent, cholangiography shows bile flow into the jejunal limb through the hepaticojejunostomy.

**Video 1** Antegrade partial stent-in-stent deployment is performed through dual ESCRs for disconnected B2 and B3 ducts after right hepatectomy with hepaticojejunostomy.


To the best of our knowledge, this is the first report of antegrade partial stent-in-stent deployment through dual ESCRs for disconnected B2 and B3 ducts after right hepatectomy with hepaticojejunostomy.

Endoscopy_UCTN_Code_TTT_1AS_2AH

## Usage rights

Figure 3 and the video were created entirely by the authors. No third-party images, icons, templates, BioRender, Flaticon, or similar external materials were used. To the best of our knowledge, no third party holds any rights of use for these materials.
